# Nurses’ Experiences, Employment Decisions and Support Needs in Nonpermanent Roles: A Scoping Review

**DOI:** 10.1155/jonm/4509202

**Published:** 2026-10-28

**Authors:** Anthony Thomas Snow, Lyn Taylor, Thomas Aaron Ricks

**Affiliations:** ^1^ School of Nursing, Midwifery and Paramedicine Australian Catholic University Fitzroy Victoria Australia acu.edu.au; ^2^ Sir Charles Gairdner Hospital Hospital Avenue Nedlands Western Australia Australia scgh.health.wa.gov.au; ^3^ Nurse Workforce Unit St Vincent’s Hospital Melbourne Fitzroy Victoria Australia svhm.org.au

**Keywords:** casual, employment, health facilities, health policy, health workforce, nurse, nursing, travel nursing

## Abstract

**Aim(s):**

To identify factors influencing nurses’ decisions to engage in nonpermanent work, examine their reported professional experiences, and map organisational support needs across non‐permanent employment arrangements.

**Background:**

Healthcare systems increasingly use nonpermanent nurses to maintain service capacity amid persistent workforce shortages and fluctuating staffing demands. Evidence on nurses’ employment decisions, professional experiences and organisational support needs remains dispersed across nonpermanent employment arrangements and healthcare settings.

**Evaluation:**

This scoping review followed Joanna Briggs Institute guidance. Five electronic databases, CINAHL Complete, MEDLINE Complete, APA PsycINFO, Scopus and Health Source: Nursing/Academic Edition, were searched in November 2025 and updated in October 2026. Eligible English‐language empirical studies examined qualified nurses’ experiences in nonpermanent roles across healthcare settings. Extracted textual findings from qualitative, quantitative and mixed methods studies were synthesised using inductive qualitative content analysis.

**Key Issue(s):**

Thirty‐one studies were included, comprising 13 quantitative, 16 qualitative and two mixed methods studies. Five analytic categories addressed professional development and career impact; employment motivations; belonging, recognition and identity; workplace constraints and psychological burden; and organisational support needs. Nurses valued flexibility, financial benefits and varied clinical experience, while reporting employment insecurity, inconsistent orientation and support, exclusion and restricted access to professional development.

**Conclusion(s):**

Nonpermanent nursing offers valued flexibility alongside variable professional experiences and organisational support. The review identifies recurring support needs but does not establish the effectiveness of specific organisational interventions.

**Implications for Nursing Management:**

Organisational priorities identified across the evidence include structured, context‐specific orientation, clear communication and escalation pathways, accessible clinical support, professional recognition and equitable access to feedback and development opportunities.

## 1. Introduction

The World Health Organization suggests a modest easing of the global nursing shortage, which decreased from 6.2 million in 2020 to 5.8 million in 2023 and is projected to decline to 4.1 million by 2030 [1]. Despite projected growth in the global nursing workforce to approximately 36 million by 2030, substantial shortages and geographic inequities are expected to persist [1]. At the same time, healthcare systems continue to face strain due to rising patient acuity, increasing service demand, population ageing and the growing burden of chronic and complex illness [2]. Compounding these pressures are escalating job demands, inadequate staff‐to‐patient ratios, limited organisational support and an ageing nursing workforce [3–5]. Loss of nursing workforce capacity places significant financial and operational pressure on health services, reduces system capability and intensifies staffing gaps [6, 7]. Without decisive action, the nursing shortage will continue to deepen, undermine progress toward universal health coverage and weaken the long‐term sustainability of healthcare systems [1, 8, 9].

Recent data indicate that nearly one in five nurses are aged 55 years or older and approaching retirement [1]. However, anticipated attrition related to workforce ageing represents only a portion of nursing turnover, as most nurses leave for reasons that organisations are able to modify [1, 10–12]. Organisational factors are the strongest predictors of nurses’ decisions to stay or leave, accounting for nearly 60 percent of turnover influences [12]. Besides planned retirement, nurses frequently exit employment in response to burnout, inability to maintain work‐life balance, lack of career advancement opportunities and family obligations [13], with burnout remaining widespread across healthcare settings and occurring at particularly high rates in high‐acuity areas [14]. Factors contributing to clinician burnout arise primarily from organisational conditions, including excessive workload, inadequate staffing, inflexible scheduling, poor employment relations, poor communication and limited organisational support [11, 15, 16]. Burnout is increasingly understood as a systems‐level outcome of sustained imbalances between job demands and available resources, requiring organisational and system‐level interventions rather than individual‐focused strategies alone [12, 17].

The terminology used to describe nonpermanent nurses varies widely across the literature. Common terms include agency nurses, travel nurses, temporary or casual nurses, float or relief‐pool nurses, per‐diem nurses and pro re nata nurses, as well as nurses employed through supplementary or gig‐based arrangements [18–23]. For this review, “nonpermanent nurse” is used as an overarching descriptor for qualified nurses working in temporary, contingent or supplementary employment arrangements rather than a permanent substantive role. Qualified nurses were defined as those holding a recognised professional nursing qualification or registration permitting clinical practice within the jurisdiction of the study, including registered nurses and enrolled nurses or international equivalents.

Health systems have increasingly relied on nonpermanent staffing models to maintain service continuity and operational flexibility amid persistent workforce shortages [24, 25]. Nonpermanent nurses contribute substantially to healthcare delivery by responding to fluctuating patient demand and supporting core services during staffing shortfalls [24, 26–28]. However, the central challenge lies not in the utilisation of nonpermanent nurses, but in the limited structural support provided, including inconsistent onboarding and suboptimal professional experiences [28]. If left unaddressed, such conditions increase the likelihood of delayed or compromised care and higher rates of clinical incidents affecting patient outcomes [14, 27, 29], such as medication errors, patient falls and nosocomial infections [14].

As health systems increasingly depend on nonpermanent nurses, understanding how these work arrangements influence job satisfaction, motivation and professional sustainability has become essential to inform workforce retention strategies, organisational support models and policy responses that are fit for contemporary staffing realities [23]. However, workforce research and policy frameworks remain largely oriented toward permanent employment models, and limited attention to healthcare workers’ lived experiences has constrained workforce policy effectiveness and contributed to fragmented evidence on the experiences and support needs of nonpermanent nurses [9, 23]. These workforce governance gaps, particularly the limited integration of healthcare workers’ lived experiences into policy design and implementation, continue to undermine effective workforce decision‐making [9]. For nurse leaders, this highlights the importance of understanding the realities of healthcare workers including nonpermanent nurses whose experiences remain largely absent from organisational decision‐making.

Previous reviews have focused predominantly on the experiences of agency registered nurses [30, 31], with less attention given to the broader range of nonpermanent nursing arrangements. This review extends the existing evidence by synthesising findings across diverse nonpermanent employment models and by examining employment decisions, professional experiences and organisational support needs together. Accordingly, this scoping review aimed to synthesise evidence on how nurses experience and describe their decisions to pursue nonpermanent employment across healthcare settings. The review addressed the following objectives:

i. Explore factors influencing nurses’ decisions to engage in nonpermanent work.
ii. Understand the benefits, challenges and broader impacts that nonpermanent nursing positions have on job satisfaction, professional identity and career development.
iii. Identify the organisational support structures and policy requirements necessary for nonpermanent nurses to maintain professional competence, safety and engagement.

## 2. Methods

### 2.1. Design

This scoping review adhered to the Preferred Reporting Items for Systematic reviews and Meta‐Analyses extension for Scoping Reviews (PRISMA‐ScR) guidelines [32]. The review protocol was registered with the Open Science Framework (link: https://doi.org/10.17605/OSF.IO/XAP4Q) and followed Peters et al. [33] nine‐step approach for conducting scoping reviews.

### 2.2. Eligibility Criteria

The eligibility criteria for this scoping review were developed using the Population, Phenomenon of Interest, Context (PICo) framework [34] and applied consistently during study selection. Included studies involving (1) qualified nurses, employed in any nonpermanent roles; (2) explored nurses’ subjective or lived experiences including career motivations, professional identity, workplace challenges or support needs; (3) were situated in any health care or aged‐care setting and (4) used empirical qualitative, quantitative or mixed methods designs.

Studies were excluded if they (1) focused solely on student nurses, nurse managers, midwives, paramedics, nursing assistants or nursing aides; (2) reported only workforce metrics or system‐level outcomes without data relating to nurses’ experiences, perceptions, employment decisions or support needs; (3) examined nonclinical nursing contexts such as administrative, educational or research‐only roles; (4) were nonempirical publications, including literature reviews, editorials, dissertations, conference abstracts, book chapters or grey literature or (5) were not published in English.

### 2.3. Sources of Evidence

Five databases were selected for their complementary disciplinary coverage. Cumulative Index to Nursing and Allied Health Literature (CINAHL) Complete (EBSCOhost) and Health Source: Nursing/Academic Edition (EBSCOhost) provided nursing and professional‐practice literature; Medical Literature Analysis and Retrieval System Online (MEDLINE) Complete (EBSCOhost) captured biomedical and health‐policy research; American Psychological Association (APA) PsycINFO (EBSCOhost) captured psychological and behavioural research relevant to motivation and work experience; and Scopus (Elsevier) provided broader multidisciplinary, organisational and workforce coverage. Together, this complementary disciplinary coverage was considered sufficient for the scope of the review. The initial and updated searches yielded a combined total of 889 records, of which 536 remained after deduplication (see Figure 1).

**FIGURE 1:**
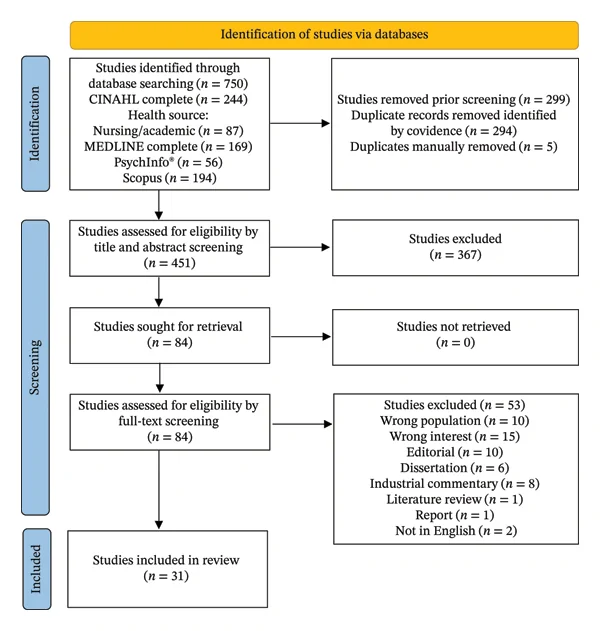
PRISMA flow diagram.

### 2.4. Search Strategy

Search terms were developed using the PICo framework. Population terms referred to qualified nurses in nonpermanent roles (e.g., “nurs∗, “casual nurse”, “agency nurse”, “non‐permanent staff”). The phenomenon of interest keywords included subjective experiences regarding their employment (e.g., “experienc∗”, “challeng∗”, “Career Progress∗”), and the context keywords included any healthcare setting from any clinical specialty (e.g., “hospital”, “aged care”, “intensive care unit”, “clinic”). No restriction was placed on the date of publication (see supporting information 1).

### 2.5. Data Selection and Collection

The databases were searched in November 2025 and then updated on 12 October 2026. Across the initial and updated database searches, 889 records were identified. Records were imported into Covidence systematic review software [35] for deduplication and screening. Following removal of 353 duplicate records, 536 unique records underwent title and abstract screening. Title and abstract screening and subsequent full‐text eligibility assessment were undertaken independently by two reviewers (AS and TR). Disagreements at either stage were resolved through discussion, with adjudication by a third reviewer (LT) where consensus could not be reached. Eighty‐four reports were retrieved for full‐text assessment, of which 53 were excluded due to wrong population (*n* = 10), wrong phenomenon of interest (*n* = 15), editorial (*n* = 10), dissertation (*n* = 6), industry commentary (*n* = 8), literature review (*n* = 1), report (*n* = 1) and publication in a language other than English (*n* = 2). Thirty‐one studies were included in the review (see Figure 1).

### 2.6. Data Charting and Extraction

Data charting followed JBI methodological guidance for scoping reviews [33, 36] and used a customised Microsoft Excel form. The form was piloted by AS using four included studies and subsequently reviewed with TR and LT to assess whether the fields were clear and captured the information required to address the review objectives. AS then charted study characteristics including author and year, country and setting, study aim, methodology, the nonpermanent nursing sample and key findings relevant to the review objectives. All completed charting was checked by a second reviewer (TR or LT), with queries discussed at regular review meetings and resolved through discussion.

Data extraction was guided by relevance to the review objectives rather than restricted to a single section of each article [33]. Relevant material was drawn primarily from the results or findings sections, with abstracts and discussion sections also examined to clarify or contextualise the reported empirical findings [36]. Material included in the synthesis had to be traceable to reported study data or data‐grounded qualitative findings. Additional source‐author explanations and recommendations were distinguished from empirical findings and were not used independently to support synthesis categories. Where studies included both eligible and ineligible participant groups, only findings attributable to eligible nonpermanent nurses were extracted for synthesis; comparator‐group data were retained only where necessary to interpret within‐study quantitative comparisons. AS and TR initially extracted data from four studies collaboratively to establish a shared understanding of the extraction approach. LT was designated as adjudicator for unresolved disagreements; however, all differences were resolved through discussion and adjudication was not required. AS subsequently extracted the relevant data from the remaining studies.

The included analytic dataset for this review comprised participant quotations and data‐grounded analytic findings reported by the authors of qualitative studies, together with narrative summaries of reported quantitative results relevant to the review objectives; raw numerical data were not independently reanalysed. Narrative summaries were checked against the reported results to distinguish measured outcomes and associations from proposed explanations. For mixed methods studies, relevant qualitative findings and narrative summaries of reported quantitative results were extracted and analysed in the same manner. These extracted textual findings formed the dataset for the qualitative content analysis.

### 2.7. Data Synthesis and Qualitative Content Analysis

Data synthesis was guided by JBI recommendations for the extraction, analysis and presentation of scoping review results [33, 36]. Extracted textual findings relevant to the review objectives were analysed using an inductive qualitative content‐analysis approach [37]. This approach enabled recurring concepts across the heterogeneous evidence base to be systematically organised while maintaining a clear link to the findings reported in the included studies and aligns with contemporary methodological recommendations for the analysis of textual findings within scoping reviews [38]. Coding focused on empirical findings relevant to the review objectives, while analytic categories were developed inductively from the extracted findings. A study contributed to a category only where it provided relevant empirical findings; source‐author explanations and recommendations alone were not counted as category contributions.

Analysis proceeded through an iterative process of coding, comparison and category development across the extracted textual findings. Relevant textual material was coded in NVivo 15 [39]. AS and TR initially coded four included studies collaboratively to refine the coding approach and clarify category boundaries. AS subsequently coded the remaining studies. Codes were iteratively compared for similarities and differences, refined where necessary and grouped into broader categories representing recurring patterns across the extracted findings. Developing categories were reviewed against the coded material and the methodological context of contributing studies and discussed with TR and LT to clarify category boundaries and maintain alignment with findings reported by the included studies. Across the analytic process, 126 preliminary codes were organised into seven broader categories and subsequently refined into five final analytic categories.

Interpretive decisions inherent in inductive category development were examined collaboratively throughout the synthesis [37]. The review team met regularly to discuss coding decisions, examine assumptions and consider alternative categorisations. As the review team comprised nurses with professional knowledge relevant to the topic, these discussions also provided an opportunity to examine assumptions that could influence categorisation.

## 3. Results

Five analytic categories were developed from the 31 included studies, comprising 13 quantitative studies [19, 40–51], 16 qualitative studies [18, 20–22, 52–63] and two mixed methods studies [23, 64]. A single aggregate participant total was not calculated because several publications drew on overlapping or nested samples, some studies did not separately report the complete eligible subgroup, and repeated responses in one study could not be linked across time points. Eligible nonpermanent nurse samples are, therefore, reported at publication level in Table 1.

**TABLE 1:** Characteristics of studies.

Author(s) and reference	Country/location	Settings	Aim	Study design	Eligible nonpermanent nurse sample^∗^	Key findings
Anderson et al. [18]	USA (Kentucky)	A single Army medical centre	Describe the perceived impact of supplemental agency nurses on patient care quality, unit cohesiveness and permanent staff morale.	Qualitative	Agency nurses (*n* = 28)^∗∗^	i. Agency nurses valued control over their work schedules, work location and choice of shift. ii. Agency nurses identified uncertainty about hours, job security and day‐to‐day work as a disadvantage of agency work. iii. Agency nurses generally perceived their contribution as reducing permanent staff workload and improving nurse–patient ratios and quality of care.

Batch and Windsor [52]	Australia (Brisbane)	A single hospital	Explore the relationship between nursing casualisation and the culture of communication within a healthcare facility.	Qualitative	Relief‐pool nurses (*n* = 9) ^∗∗^ ^,^ ^a^	i. Relief‐pool nurses described incomplete orientation, inadequate handover and limited access to patient information when entering unfamiliar units. ii. Relief‐pool nurses described anxiety and disconnection associated with working without adequate information. iii. The authors identified relief nurses as being positioned as outsiders and subject to stereotyping, with marginalisation also limiting access to learning and skill‐development opportunities.

Berg Jansson and Engström [53]	Sweden (Norrbotten County)	A single hospital ICU	Describe critical‐care nurses’ experiences of working with, or as, temporary agency staff.	Qualitative	Agency critical care nurses (*n* = 5)^∗∗^	i. Agency critical‐care nurses valued professional freedom and opportunities to work closely with patients. ii. Collaboration was more difficult when work required local organisational knowledge or familiarity with unit routines. iii. Agency nurses described less‐close collegial relationships than regular critical‐care nurses.

Berg Jansson et al. [54]	Sweden (Norrbotten County)	A single hospital ICU	Analyse conditions for workplace learning in temporary agency staffing, focussing on agency and regular nurses’ experiences of social relations.	Qualitative	Agency critical care nurses (*n* = 5)^∗∗^	i. Agency critical‐care nurses had limited participation in formal workplace‐development activities. ii. Patient‐related work and knowledge exchange with colleagues provided opportunities for informal workplace learning. iii. Less‐close social relationships with colleagues constrained opportunities for learning and integration.

Bethel et al. [40]	USA	Various healthcare settings	Identify travel nurses’ onboarding needs and describe current onboarding practices.	Quantitative	Travel nurses (*n* = 306)	i. Travel nurses prioritised facility‐ and unit‐specific orientation. ii. They preferred advance information about scheduling, patient ratios, floating expectations and other assignment requirements, together with timely access credentials. iii. Travel nurses wanted onboarding and competency processes tailored to the unit or facility in which they would work.

Braddy et al. [41]	USA (Tri‐state area)	Multiple hospitals	Understand the reasons RNs choose agency work	Quantitative	Agency nurses (*n* = 40)	i. Flexible scheduling and higher pay were prominent reasons for choosing agency work. ii. Agency nurses’ employment decisions were also influenced by perceived shortcomings in hospital employment, including fair treatment, supervision, recognition, feedback and work allocation.

Chamanga et al. [55]	England	Two community NHS trusts (urban and coastal)	Explore why community nurses with temporary contracts join an adult community NHS trust and what influences their retention.	Qualitative	Temporary community nurses (*n* = 4)	i. Temporary community nurses identified flexibility, independence and financial reward as attractions of temporary employment. ii. The three agency nurses described negative experiences including feeling exploited or unappreciated, heavy caseloads and poor communication or integration; the bank nurse described her team and organisation more positively. iii. Experiences of organisational support, team relationships and communication influenced participants’ views of remaining in temporary community nursing.

Cicellin et al. [56]	Italy (Naples)	A single hospital	Explore collective identity and resistance among temporary agency nurses within a hospital employment context.	Qualitative	Temporary agency nurses (*n* = 10)^∗∗^	i. Temporary agency nurses described insecurity and fear associated with their employment status, alongside solidarity among temporary colleagues. ii. The authors interpreted this collective identity as facilitating subtle forms of resistance to management practices. iii. The authors conceptualised temporary contract status as a dimension differentiating temporary and permanent workers.

de Ruyter [64]	UK (West Midlands, Leeds, and London)	Multiple hospitals	Explore motivations for agency nursing	Mixed methods	Agency nurses (*n* = 233)	i. Flexibility and work‐life balance were prominent reasons for agency nursing, alongside financial benefits. ii. Dissatisfaction with conditions in permanent NHS employment also influenced movement into agency work. iii. Nurses working solely through agencies placed value on flexibility and distance from workplace politics.

Faller et al. [42]	USA (Nationwide)	Multiple hospitals	Examine work‐related burnout, job satisfaction, intent to leave and nurse‐assessed quality of care among travel nurses, and their relationships with nurse and workplace characteristics.	Quantitative	Travel nurses (*n* = 976)	i. Travel nurses reported significantly higher job satisfaction and perceived quality of care in Magnet‐designated hospitals. ii. Work‐related burnout increased as the number of patients assigned to travel nurses increased. iii. Travel nurses working in California reported significantly lower work‐related burnout than those working elsewhere.

Faller et al. [57]	USA (Nationwide)	Multiple hospitals	Explore travel nurses’ motivations and challenges	Qualitative	Travel nurses (*n* = 17)	i. Reasons for travel nursing included flexibility, dissatisfaction with permanent positions, gaining experience across hospitals and opportunities to work in different locations. ii. Travel nurses described considerable variation in the orientation, inclusion and support provided by host facilities. iii. Participants identified a need for orientation focused on hospital‐ and unit‐specific policies, procedures and practical resources.

Fiore‐Lopez et al. [19]	USA (Nationwide)	Various healthcare settings	To determine differences between travel and nontravel nurses’ perceptions of their work environment and ethical work climate	Quantitative	Travel nurses (*n* = 615)^∗∗^	i. Travel nurses reported lower scores than non‐travel nurses for being valued as an integral team member and for courteous and respectful treatment from permanent co‐workers. ii. Travel nurses also reported lower perceptions of fairness in patient assignment and workload allocation. iii. Overall healthy‐work‐environment scores did not differ significantly between travel and non‐travel nurses, and neither group perceived an overall healthy work environment.

Fischer et al. [20]	Netherlands	Multiple hospitals	Explore perceived demands and resources associated with hospital float‐pool work, comparing experienced intra‐organisational float nurses with nurses anticipating inter‐organisational floating.	Qualitative	Intra‐organisational float‐pool nurses (*n* = 10)^∗∗^	i. Intra‐organisational float‐pool nurses generally reported job satisfaction. ii. Learning opportunities and variation in tasks were important job resources. iii. Reported demands included limited team acceptance and communication or terminology differences; openness, confidence and communication skills supported adaptation.

FitzGerald et al. [21]	Australia (NSW)	Regional public health service	Identify factors enabling or obstructing casual‐pool nurses’ delivery of high‐standard nursing care within multidisciplinary teams.	Qualitative	Casual pool nurses (*n* = 61)	i. Balancing social and professional life was a central feature of casual‐pool nurses’ experience and was facilitated by employment flexibility. ii. Casual‐pool nurses described limited feedback, difficulties integrating into unfamiliar teams and challenges navigating local workplace routines and politics. iii. Nurses considered their experience across different units to provide useful insight into clinical environments and team cultures.

Han et al. [43]	South Korea	Multiple hospitals	Compare empowerment, job satisfaction and organisational commitment between temporary and permanent nurses	Quantitative	Temporary nurses (*n* = 162)^∗∗^	i. Temporary nurses reported significantly lower job satisfaction, organisational commitment and psychological empowerment than permanent nurses. ii. Temporary nurses reported significantly lower scores on the perceived competence subscale of psychological empowerment than permanent nurses.

Hansen and Tuttas [58]	USA (Nationwide)	Multiple hospitals	Explore the pandemic’s impact on nurse motivation, happiness, stress and career decisions	Qualitative	Travel nurses (*n* = 20: 10 veteran, 10 new)^∗∗^	i. Among new travel nurses, higher hourly pay was the most frequently cited reason for entering travel nursing, although purpose‐driven motives were also reported. ii. New travel nurses described working across different hospitals as strengthening their competence, expanding their skills and increasing confidence. iii. Veteran travel nurses described pandemic‐related physical and emotional trauma and were more likely to consider leaving bedside nursing or their current role. iv. Half of the travel nurses reported being treated as outsiders; some also described bullying by permanent staff and being given the heaviest assignments.

Hass et al. [59]	UK (London)	A single hospital ICU	Explore ICU agency nurses’ lived experience	Qualitative	Agency nurses (*n* = 8)	i. Agency ICU nurses described lacking confidence, deskilling and isolation. ii. Participants identified orientation, clinical support, feedback and continuing professional development as important needs.

Hermes et al. [44]	Germany; recruitment extended to German‐speaking regions of Austria, Switzerland, Belgium, Luxembourg, Liechtenstein and Italy	Multiple hospitals; intensive, intermediate and special care units.	Examine job satisfaction, working conditions and perceptions of agency work among nurses in intensive, intermediate and special care.	Quantitative	Agency nurses (*n* = 154)^∗∗^	i. Agency nurses valued variety and new challenges; 76% reported that opportunities to gain experience across different areas would be important if considering a return to permanent employment. ii. Almost half of agency nurses completing the agency‐specific questions reported receiving no onboarding at deployment hospitals, and many reported being expected to use equipment without appropriate instruction. iii. Agency nurses generally perceived their contribution positively: 90% considered agency nurses valued, 93% believed they made work easier for regular staff and 75% believed their deployment improved job satisfaction on the unit.

Hickey et al. [60]	USA (Massachusetts)	A single hospital	Explore the experience and perceived value of paediatric travel nurses in a children’s hospital.	Qualitative	Paediatric travel nurses (*n* = 56)	i. Financial incentives and scheduling flexibility were prominent features of paediatric travel nursing. ii. Participants described unhealthy work environments, short staffing, burnout and poor leadership as reasons for seeking travel assignments and healthier workplaces. iii. Travel nursing provided opportunities to gain experience and skills across settings, although some participants also described isolation, depression and uncertainty between contracts.

Hughes and Marcantonio [45]	USA (Illinois)	Various healthcare settings	Examine recruitment‐, retention‐ and compensation‐related differences between agency and hospital staff nurses.	Quantitative	Agency nurses (*n* = 3360)^∗∗^	i. Agency nurses received higher hourly wages but fewer employment benefits than hospital nurses. ii. Agency and hospital nurses differed in the employment incentives associated with changing jobs and increasing workload.

Jalonen et al. [46]	Finland	Multiple hospitals	Determine demographic and work‐related predictors of sustained organisational commitment among temporary nurses and examine the effect of transition to permanent employment.	Quantitative	Temporary nurses (*n* = 412)	i. Older age, higher job control, greater participative safety, greater procedural justice and lower psychological distress predicted sustained organisational commitment among temporary nurses. ii. In the fully adjusted model, transition from temporary to permanent employment and higher job control remained significant predictors of sustained organisational commitment; older age also remained significant.

Klumb et al. [47]	Switzerland	Multiple hospitals	Examine how responsibility and social appreciation shape pay‐unfairness evaluations and their associations with exhaustion and job satisfaction among temporary and staff nurses.	Quantitative	Temporary nurses (*n* = 120)^∗∗^ • Travel nurses (*n* = 55) • Per‐diem nurses (*n* = 59) • Unspecified temporary nurses (*n* = 6)	i. The negative association between perceived pay unfairness and job satisfaction was stronger among temporary nurses than staff nurses; no comparable group difference was found for exhaustion. ii. Follow‐up analyses indicated that temporary nurses treated social appreciation as an employment outcome rather than as a reference point for judging pay fairness. iii. Among temporary nurses, greater patient appreciation was associated with lower perceived pay unfairness and attenuated the association between pay unfairness and exhaustion.

Manias et al. [61]	Australia (Melbourne)	Multiple hospitals	Gain an in‐depth understanding of agency nurses’ clinical practice, relationships with employing agencies, hospitals and permanent nurses, and professional status.	Qualitative	Agency nurses (*n* = 10)	i. Flexibility was the most frequently identified reason for undertaking agency nursing. ii. Short‐notice work, uncertainty about allocations and inconsistent orientation could create anxiety when entering unfamiliar settings. iii. Agency nurses described concerns relating to professional isolation, continuing education and maintenance or development of clinical skills.

Owens et al. [62]	USA (Maryland)	Multiple hospitals	Explore factors associated with travel nurses completing or extending an assignment or leaving before assignment completion.	Qualitative	Travel nurses (*n* = 14)	i. Flexibility, support and assignment conditions were the three principal themes identified in travel nurses’ accounts. ii. Travel nurses reported being more willing to complete or extend assignments when they felt supported, retained flexibility and found the assignment location desirable. iii. Support from host‐facility staff and managers was important to participants’ satisfaction with assignments.

Prieto‐de Benito et al. [48]	Spain (Nationwide)	Multiple hospitals	Examine associations between employment instability, burnout, humanised care and work engagement, including a serial mediation model.	Quantitative	Fixed‐term nurses (*n* = 400)	i. A greater number of temporary contracts was associated with higher burnout and lower work engagement. ii. Burnout was negatively associated with work engagement and dimensions of perceived humanised care. iii. Mediation analyses identified indirect statistical pathways linking employment instability, burnout, humanised care and work engagement.

Raso and Fitzpatrick [49]	USA (Nationwide)	Various healthcare settings	Examine travel nurses’ perceptions of work environment, authentic leadership, wellbeing and pandemic impact	Quantitative	Travel nurses (*n* = 56)^∗∗^	i. Travel nurses perceived authentic nurse leadership as present most of the time. ii. They did not perceive an overall healthy work environment: five of six healthy‐work‐environment domains were rated as absent, with skilled communication the only domain rated as present. iii. Pandemic impact on practice was high (mean 8.1/10), and 70% of travel nurses had wellbeing scores in the “at‐risk” range.

Ronnie [63]	South Africa	A single hospital ICU	Examine agency nurses’ ICU experiences and interprofessional relationships	Qualitative	Agency nurses (*n* = 9: Enrolled 7, Registered 2)^∗∗^	i. Eligible registered and enrolled nurses described challenges associated with inconsistent or haphazard clinical allocation and feelings of exclusion. ii. Experiences of support varied, with some describing practical and interpersonal support from permanent staff and unit managers.

Sharrock et al. [22]	Australia (Melbourne)	Major metropolitan health service	To explore workplace satisfaction for nurses employed in the supplementary employment models.	Qualitative	Bank nurses (*n* = 7)^∗∗^ ^,^ ^b^	i. Bank nurses valued flexibility and the ability to choose shifts around personal and family commitments; however, cancelled or non‐guaranteed shifts could create uncertainty in work and income. ii. All Bank nurses identified limited access to education and professional‐development opportunities, although working across different wards also provided opportunities for experiential learning and skill development. iii. Bank nurses described variable experiences of ward integration and support, ranging from feeling separate from regular ward staff to feeling welcomed and supported in individual clinical areas.

Tan et al. [23]	Singapore (Nationwide)	Various healthcare settings	To examine temporary registered nurses’ job satisfaction, motivation, intent to stay, and work experiences in gig or locum roles.	Mixed methods	Temporary nurses (*n* = 111)	i. Higher compensation, scheduling flexibility and autonomy were prominent attractions of gig and locum nursing. ii. Intrinsic job‐satisfaction scores were higher than extrinsic scores; career advancement was the lowest‐rated satisfaction item. iii. Intention to stay was low, while interviews identified limited opportunities for clinical and professional growth as an important challenge.

Yeh et al. [50]	Taiwan (Northern Taiwan)	Multiple hospitals	Compare job stress, affective organisational and occupational commitment, and perceived psychological‐contract breach between temporary and permanent nurses.	Quantitative	Temporary nurses (*n* = 120) ^∗∗^	i. Temporary nurses reported significantly greater job stress and lower affective organisational and occupational commitment than permanent nurses. ii. Temporary and permanent nurses did not differ significantly in their perceptions of psychological contract breach. iii. Greater perceived psychological contract breach was associated with higher job stress and lower affective organisational and occupational commitment; the associations with job stress and occupational commitment were stronger among permanent than temporary nurses.

Young et al. [51]	USA (Ohio)	A single hospital	Evaluate the Parent Shift Pro re nata (PRN) programme, including nurses’ motivations, incentives, roles and orientation	Quantitative	PRN nurses (*n* = 83 hired)^∗∗^ ^,^ ^c^	i. PRN nurses valued the programme’s short shifts and flexibility in selecting working times. ii. Flexible scheduling supported nurses’ ability to participate in paid nursing work while accommodating other personal and family responsibilities. iii. The programme included experienced nurses returning to clinical nursing after extended periods away from practice.

Abbreviations: ICU = intensive care unit, NHS = National Health Service, PRN = pro re nata, RN = registered Nurse, UK = United Kingdom, USA = United States of America.

^∗^All included sample sizes are reported by publication and should not be summed to derive a unique participant total because some publications draw on overlapping or nested samples, some studies did not separately report the complete eligible subgroup, and some repeated survey responses could not be linked.

^∗∗^Only participants meeting the review eligibility criteria are reported in the sample column where included studies contained mixed participant populations.

^a^Batch and Windsor [52] reported nine relief‐pool nurses among the individual interview participants; additional relief‐pool nurses participated in focus groups, but the exact number of eligible relief‐pool participants in those groups was not separately reported.

^b^Sharrock et al. [22] reported themes across a combined sample of seven casual bank nurses and three permanently employed pool nurses. Only findings explicitly attributable to bank nurses are reported in this review.

^c^Young et al. [51] reported 83 parent shift nurses hired during the study period; 34 completed the survey at programme entry and 55 completed the 4‐month follow‐up survey. Surveys were anonymous and responses could not be linked across time points.

The evidence base was geographically dispersed but concentrated in the United States and Europe, with the 31 studies representing 14 primary country locations. The United States contributed 12 studies, accounting for 38.7% of the evidence base [18, 19, 40–42, 45, 49, 51, 57, 58, 60, 62]. Eleven studies originated from eight European countries: Sweden [53, 54], Italy [56], the United Kingdom [55, 59, 64], Germany [44], Finland [46], Switzerland [47], the Netherlands [20] and Spain [48]. Four studies were conducted in Australia [21, 22, 52, 61], three in Asia, comprising South Korea [43], Singapore [23], and Taiwan [50], and one in South Africa [63]. One German study also recruited participants from German‐speaking regions of neighbouring European countries [44]. Overall, the geographical distribution was broad but uneven, with most studies originating from North America, Europe and Australia (Table 1).

### 3.1. Category 1: Professional Development and Career Impact

Thirteen studies contributed to this category [20–23, 43, 51, 52, 54, 57–61]. This category captures findings relating to access to formal and informal learning, maintenance and development of clinical skills, feedback and appraisal, professional confidence, career progression and movement into or through different nursing roles.

Nonpermanent nurses described opportunities to develop knowledge and skills through exposure to different clinical environments. Working across hospitals, wards and patient populations provided opportunities to encounter different practices and broaden clinical experience [20, 22, 57, 60, 61]. New travel nurses similarly described working across different hospitals as strengthening competence, expanding skills and increasing confidence [58]. Han et al. [43] found that temporary nurses reported significantly lower scores on the perceived competence subscale of psychological empowerment than permanent nurses. Experiences were not uniformly positive, however. Agency nurses in Manias et al. [61] described differing views of career development: Some regarded varied clinical exposure as beneficial to their skills and professional development, whereas others considered task‐focused agency work to offer fewer opportunities to develop knowledge or progress professionally.

Across included studies, access to formal professional development was often limited, with nonpermanent nurses describing restricted access to in‐service education, educator support, feedback and structured development opportunities [21, 22, 52, 59, 61]. Learning was nevertheless described through varied clinical exposure, patient care and knowledge exchange with colleagues [20, 22, 54, 61]. Agency nurses in Manias et al. [61] also described individual responsibility for maintaining their own professional development. Some studies further described reduced opportunities to participate in learning and skill‐development activities when nonpermanent nurses occupied a peripheral position within established clinical teams [52, 54]. These findings are consistent with the primary studies’ descriptions of limited ongoing education, deskilling concerns, individual responsibility for maintaining education and workplace learning through social interaction and knowledge exchange.

FitzGerald et al. [21] and Tan et al. [23] reported restricted opportunities for professional advancement or growth. Nonpermanent roles also featured in different forms of career transition. Some casual‐pool nurses used the breadth of experience gained across clinical areas before subsequently moving into permanent employment [21]. The Parent Shift programme, by comparison, provided a flexible route into clinical work for experienced nurses, including nurses returning after periods away from hospital nursing [51].

Travel nursing studies described professional growth alongside changes in career intentions. Participants reported gaining experience and skills through exposure to different patient populations and healthcare settings, while new travel nurses described increased competence, broader skills and greater confidence from working across hospitals [58, 60]. In contrast, veteran travel nurses described pandemic‐related physical and emotional trauma, with more participants in this group expressing intentions to leave bedside nursing or their current role [58]. Across the studies contributing to this category, nonpermanent nursing roles provided opportunities for experiential learning, skill development and career transition, while access to formal education, feedback and structured career progression was variable and, in several settings, limited.

### 3.2. Category 2: Motivations for Choosing Nonpermanent Work

Sixteen studies contributed to this category [18, 20–23, 41, 44, 45, 51, 55, 57, 58, 60–62, 64]. This category captures findings relating to nurses’ reasons for choosing, preferring or continuing in nonpermanent work, including flexibility and control over working arrangements, financial considerations, alignment with personal circumstances, opportunities for varied experience and aspects of permanent employment that influenced movement towards nonpermanent roles.

Scheduling flexibility and control over working arrangements were recurrent reasons for choosing or preferring nonpermanent work. Nurses valued greater choice over when and where they worked, including the ability to select shifts and accommodate responsibilities outside employment [18, 21–23, 41, 61, 64]. In Anderson et al. [18], agency nurses described positively the control they had over their schedules, work locations and shift selection. Flexibility also enabled nurses to accommodate family responsibilities, study, other employment and changes in personal circumstances [20, 21, 51]. FitzGerald et al. [21] found that the ability to choose shifts was the most reported reason for casual‐pool work, while Fischer et al. [20] described schedule flexibility as a valued resource, including for a nurse whose parenting responsibilities were incompatible with weekday night shifts.

Financial considerations were prominent in several studies but were not uniformly decisive in nurses’ employment choices. Higher pay or financial incentives featured in decisions to undertake agency, travel and other nonpermanent work [23, 41, 45, 58, 60]. Braddy et al. [41] identified flexible scheduling and higher pay among commonly reported reasons for agency work, although participants also valued fair treatment, supervision, recognition and feedback. Higher remuneration was similarly prominent among travel nurses, particularly during the COVID‐19 pandemic [58, 60]. In contrast, Manias et al. [61] reported that financial remuneration was not identified by participants as a reason for undertaking agency work, while travel nurses in Owens et al. [62] described compensation as a benefit rather than the primary reason for beginning or continuing travel nursing.

Variety in clinical work and exposure to different settings were additional motivations for entering or preferring nonpermanent roles. Travel nurses described opportunities to gain experience across hospitals, encounter different procedures and practices, work in different locations and explore alternative workplaces [57, 60]. Float‐pool nurses similarly valued learning opportunities and exposure to different departments [20], while agency nurses in Hermes et al. [44] expressed preferences for diversity and new challenges rather than consistency within a single team. Temporary community nurses identified flexibility, independence, autonomous practice, location, financial reward and personal choice among factors relevant to their employment arrangements [55].

Dissatisfaction with aspects of permanent employment was explicitly reported as a reason for moving towards nonpermanent work. Participants described inflexible rostering, workplace politics, stressful work environments, inadequate staffing and limited leadership support in relation to their employment decisions [21, 57, 58, 60, 61, 64]. Casual‐pool nurses in FitzGerald et al. [21], for example, valued remaining outside the workplace politics associated with permanent teams. Hansen and Tuttas [58] reported that perceived lack of leadership support and hospital politics influenced some nurses’ decisions to enter travel nursing, while Hickey et al. [60] described inadequate staffing, burnout, poor leadership and the search for healthier work environments among participants’ reasons for leaving permanent positions for travel nursing.

For nurses already working in nonpermanent roles, flexibility, assignment characteristics and workplace support influenced decisions to continue or renew these arrangements. Travel nurses identified organisational support as an important consideration when deciding whether to renew assignments and continue travelling [62]. Bank nurses similarly valued flexibility and control over shift choice [22], while gig‐based nurses identified compensation, flexibility and autonomy among valued features of this mode of employment [23]. Nonpermanent arrangements also enabled some nurses to remain engaged in nursing alongside further study, other employment or periods of workforce re‐entry [21, 51]. Across the studies, nurses identified scheduling flexibility and control, financial considerations, varied clinical experience and compatibility with personal circumstances as reasons for choosing or continuing nonpermanent work, while dissatisfaction with particular aspects of permanent employment also influenced movement towards these roles.

### 3.3. Category 3: Belonging, Recognition and Identity

Seventeen studies contributed to this category [19–22, 44, 47, 52–56, 58–63]. This category captures findings relating to nonpermanent nurses’ inclusion within clinical teams, recognition and respect for their professional contribution, relationships with permanent colleagues and experiences of professional positioning and identity associated with their employment status.

Marginalisation and limited integration within established clinical teams characterised the experiences of nonpermanent nurses in several settings. Relief‐pool and agency nurses described being positioned at the periphery of teams, developing less‐close relationships with permanent colleagues or experiencing difficulty establishing a sense of belonging [22, 52–54, 59, 63]. Float‐pool nurses similarly described limited team acceptance and a sense of not fully belonging despite otherwise positive interactions with colleagues [20]. Tensions between nonpermanent and permanent nurses were also reported in travel‐nursing contexts, including animosity during the COVID‐19 pandemic and less favourable relationships with permanent staff in some assignments [58, 60, 62].

Recognition of professional contribution and respectful treatment also differed across nonpermanent employment contexts. Nurses described experiences of being undervalued, unappreciated or treated differently because of their employment arrangement, although these experiences varied between settings and teams [22, 52, 55, 61–63]. Fiore‐Lopez et al. [19] found no meaningful overall difference between travel and nontravel nurses’ perceptions of the work environment, but travel nurses reported lower ratings for being treated with courtesy and respect by coworkers and for being valued as an integral member of the nursing team. Klumb et al. [47] further found that temporary nurses incorporated social appreciation from colleagues and patients into evaluations of their employment exchange and perceived pay fairness.

Professional identity was shaped in some studies by the distinction between nonpermanent and permanent employment status. Batch and Windsor [52] described stereotyping and marginalising practices that positioned nonstandard nurses as outsiders, including assumptions about their competence and commitment. Cicellin et al. [56], through their qualitative analysis, linked experiences of fear with solidarity and collective identity among temporary agency nurses. Agency and temporary nurses in other studies similarly described distinctions between themselves and permanent staff in relation to team membership, acceptance and workplace relationships [61, 63].

Positive experiences of belonging were associated with collegial support, team acceptance and acknowledgement of nurses’ contribution. Agency nurses described occasions when permanent staff were welcoming, friendly and supportive, while float‐pool nurses identified appreciation from leaders, teams, organisations and patients as a positive feature of their work [20, 61]. Positive and negative experiences of workplace support were likewise reported among temporary community, travel and bank nurses, with participants describing supportive relationships in some settings and resentment, poor integration or limited acceptance in others [22, 55, 62]. Hermes et al. [44] also reported that agency nurses could be excluded from information flows and identified respect and appreciation as relevant features of their workplace experiences. Across the studies contributing to this category, nonpermanent nurses experienced both inclusion and exclusion within clinical teams, with findings encompassing collegial support and professional recognition as well as marginalisation, weaker team relationships, differential treatment and outsider positioning.

### 3.4. Category 4: Workplace Constraints and Psychological Burden

Twenty‐three studies contributed to this category [18–22, 42–44, 46–50, 52, 53, 55, 56, 58–63]. This category captures findings relating to workplace and employment conditions that constrained nonpermanent nursing practice, including unfamiliar clinical environments, assignment and workload practices, rostering and contractual uncertainty, employment insecurity and reported psychological and work‐related outcomes.

Frequent movement between clinical environments required nonpermanent nurses to adapt to unfamiliar routines, procedures, information systems and workplace expectations. Agency, float‐pool and temporary nurses described differences in local routines, protocols and working practices that had to be navigated when moving between units or organisations [20, 53, 61, 62]. Inconsistent access to local information and guidance was also reported, including exclusion from information flows and, in some cases, limited instruction in the use of equipment or local procedures [22, 44, 61, 63]. Travel nurses in Owens et al. [62] similarly described differences in policies, procedures and unit organisation between assignments as challenging aspects of travel work.

Clinical allocation, workload and rostering practices were sources of uncertainty or dissatisfaction in several nonpermanent nursing settings. Nurses described short‐notice or unclear allocations, variable caseloads and uncertainty about where or how they would be deployed [21, 22, 55, 61, 63]. Fiore‐Lopez et al. [19] found that travel nurses gave lower ratings than nontravel nurses to receiving patient workloads or assignments that were fair and consistent with those of coworkers, although this did not establish that travel nurses routinely received heavier assignments. Hansen and Tuttas [58] reported that some travel nurses perceived that they received the heaviest or less desirable assignments during the COVID‐19 pandemic. Workload was also quantitatively associated with work‐related burnout among travel nurses in Faller et al. [42], with burnout scores increasing as the number of assigned patients increased.

Agency and temporary nurses described uncertainty regarding future work, continuity of employment and contractual stability [18, 22, 56, 60]. Travel nurses also described uncertainty about securing subsequent positions, licensing requirements and employment benefits [60, 62]. Perceived pay unfairness was also associated with lower job satisfaction and greater exhaustion in Klumb et al. [47], with the association between pay unfairness and job satisfaction stronger among temporary than staff nurses; the relationship with exhaustion did not differ significantly between the groups.

Psychological strain was reflected in reports of anxiety, fear, isolation, emotional trauma and compromised wellbeing. Relief‐pool and agency nurses described anxiety or reduced confidence when working with incomplete information, unfamiliar environments or uncertain clinical responsibilities [52, 59, 61]. Temporary workers in Cicellin et al. [56] described insecurity and fear associated with their employment circumstances, while travel nurses reported emotional and psychological strain arising in different contexts, including pandemic‐related trauma and prolonged separation from family [58, 60]. Hickey et al. [60] specifically reported feelings of isolation and depression among some travel nurses undertaking sequential assignments away from their families. Quantitative findings similarly identified wellbeing concerns: Raso and Fitzpatrick [49] reported that 70% of travel nurses in their sample had Well‐Being Index scores classified as at risk, alongside a high reported impact of the COVID‐19 pandemic on practice.

Less favourable work‐related outcomes, including higher burnout and job stress, lower job satisfaction and work engagement, reduced psychological empowerment and lower organisational or occupational commitment, were identified across several quantitative studies of nonpermanent nurses, although the specific constructs and employment conditions examined differed. Higher patient load was associated with higher work‐related burnout scores among travel nurses [42], while temporary nurses reported lower job satisfaction, organisational commitment and psychological empowerment than permanent nurses [43]. Higher job stress and lower affective organisational and occupational commitment were also reported among temporary nurses, with psychological contract breach associated with greater stress and lower commitment [50]. Among fixed‐term nurses, greater contract instability was associated with higher burnout and lower work engagement [48]. Conversely, more favourable workplace conditions were associated with sustained organisational commitment: greater job control, participative safety and procedural justice, together with lower psychological distress, predicted sustained commitment in Jalonen et al. [46], with job control remaining significant in the fully adjusted model.

### 3.5. Category 5: Experiences of Organisational Support and Unmet Support Needs

Seventeen studies contributed to this category [20–23, 40, 44, 49, 52–55, 57, 59–63]. This category captures findings relating to the organisational resources, information, clinical guidance, leadership, workplace relationships and development opportunities available to nonpermanent nurses, together with areas in which these forms of support were reported as absent, inconsistent or insufficient.

Access to structured, setting‐specific orientation and information was a prominent support need when nurses entered unfamiliar clinical environments. Travel and agency nurses identified a need for information about unit routines, patient ratios, rostering and float policies, local procedures, equipment, electronic systems and organisational expectations [40, 57, 59, 61]. Bethel et al. [40] found that 93.9% of travel nurses preferred orientation to be specific to the facility, while participants also prioritised advance information about patient ratios and scheduling, timely system access and experience with unit routines. In qualitative studies, orientation ranged from comprehensive preparation to brief or inconsistent introductions, with agency and relief‐pool nurses sometimes beginning shifts without sufficient contextual or patient information [52, 59, 61]. Manias et al. [61] further reported that agency nurses wanted written ward‐specific information that could be retained for future reference, including local procedures, documentation requirements, emergency processes and relevant contacts.

Reliable access to clinical guidance, communication and knowledgeable colleagues was important when non‐permanent nurses worked across changing units and organisations. Nurses described the value of approachable staff, clear communication channels, accessible clinical information and identifiable colleagues to whom questions could be directed [20, 57, 59, 61, 63]. Agency nurses in Hass et al. [59] described lower anxiety when team leaders and permanent staff were friendly and approachable. Travel nurses in Faller et al. [57] described positive assignments as those in which hospitals provided appropriate orientation, consistent clinical resources, access to information and a designated buddy or resource nurse; poor experiences included lack of managerial support and restricted access to information available to permanent staff.

Interpersonal and leadership support differed substantially across settings, ranging from welcoming and accessible support to poor integration or limited managerial engagement. Temporary community, float‐pool, agency, travel and bank nurses described the quality of support from permanent colleagues, managers and host organisations as an important feature of their work experiences [20, 22, 55, 62, 63]. Agency nurses in Ronnie [63] reported practical and interpersonal support from permanent nurses and unit managers despite also describing exclusion and allocation difficulties. Raso and Fitzpatrick [49] similarly found that travel nurses perceived authentic nurse leadership to be present most of the time, although five of six healthy‐work‐environment domains were rated as absent. Among travel nurses in Owens et al. [62], support was one of three central themes and was described as a primary consideration in renewing assignments and continuing to travel.

Access to education, feedback and professional development represented another area in which organisational support was frequently limited. Casual, agency and bank nurses described reduced access to in‐service education, formal feedback and structured learning opportunities compared with permanent colleagues [21, 22, 52, 54, 59, 61]. Shift patterns and peripheral workforce positioning could also limit participation in meetings and educational activities [52], while workplace learning among agency nurses often occurred informally through patient care and knowledge exchange rather than through formal organisational development structures [54]. Limited opportunities for clinical and professional growth were also described in gig‐based nursing arrangements [23].

Support needs extended beyond orientation and clinical assistance to the broader conditions of an assignment. Travel nurses considered staffing levels, assignment expectations, unit resources and the quality of the work environment when evaluating potential or future contracts [60, 62]. Owens et al. [62] reported that nurses considered leaving assignments because of safety concerns or unsupportive environments and evaluated nurse‐to‐patient ratios and specialty fit when accepting work. Hickey et al. [60] similarly found that participants considered staffing, leadership and healthy‐work‐environment characteristics when assessing future travel assignments. Agency nurses in Hermes et al. [44] also reported gaps in onboarding and instruction, including instances of beginning work without adequate orientation or being expected to use unfamiliar equipment without instruction. Across the studies contributing to this category, organisational support was described through orientation, access to local information and clinical guidance, collegial and managerial support, education and feedback, and appropriate assignment resources; however, the availability and consistency of these supports differed substantially across employment arrangements and clinical settings.

## 4. Discussion

The findings suggest that the value of nonpermanent nursing work is shaped by both the flexibility it offers and the organisational conditions under which that flexibility is experienced. Across different employment models, nurses valued greater control over working arrangements and opportunities for varied clinical experience, while experiences of professional development, team integration, employment security and organisational support were less consistent.

The principal contribution of this review is the integration of evidence on nurses’ employment decisions with their professional experiences and organisational support needs. The present review synthesised 31 empirical studies across agency, travel, casual, bank, float‐pool, temporary, supplementary and gig or locum arrangements, showing that valued employment flexibility coexisted with inconsistent access to professional development, recognition, team inclusion and support. Nurses’ accounts also connected decisions to enter nonpermanent work with dissatisfaction with permanent employment, alongside preferences for flexibility, financial benefits and varied clinical experience. These findings suggest that understanding nonpermanent nursing requires attention both to what attracts nurses to these arrangements and to the conditions they encounter within them.

This scoping review complements Telschow and Berg’s [28] qualitative synthesis of induction and rapid orientation in hospital settings by placing orientation within a wider account of employment decisions, career development and professional experience. It also complements Wolbers et al.’s [65] realist review, which examined how temporary‐nurse staffing models generate patient, staff and health‐system outcomes in different contexts. Although organisational support is a shared concern, the present review centres nurses’ reasons for choosing or continuing nonpermanent employment alongside their experiences within these roles. Its added contribution is the integration of these dimensions across study designs and employment arrangements, identifying recurring support needs that extend beyond initial orientation.

### 4.1. Employment Decisions

Nurses’ decisions to engage in nonpermanent work reflected both dissatisfaction with conditions associated with permanent employment and the perceived advantages of more flexible working arrangements. Within the included studies, inflexible rostering, workplace politics, inadequate staffing, stressful work environments, burnout and limited leadership support were described alongside preferences for greater control over shifts, compatibility with personal circumstances, financial benefits and varied clinical experience [18, 20, 21, 41, 57, 58, 60–62, 64]. These findings indicate that movement into nonpermanent roles cannot be attributed solely to the characteristics of flexible work or dissatisfaction with permanent employment, because both were present within nurses’ accounts of their employment decisions.

Several included studies explicitly linked movement into nonpermanent nursing with dissatisfaction with aspects of permanent employment, including inflexible working arrangements, workplace politics, inadequate staffing, burnout, stressful work environments and limited leadership support [21, 57, 58, 60, 61, 64]. These findings are consistent with factors associated with turnover in the broader nursing workforce. Muir et al. [13] found that, excluding planned retirement, burnout or emotional exhaustion, insufficient staffing and family obligations were prominent contributors to nurses ending healthcare employment; among nurses who moved into employment outside healthcare, better wages, benefits or work flexibility were also frequently identified. Sasso et al. [66] similarly identified emotional exhaustion as a factor associated with intention to leave, whereas job satisfaction, leadership, participation in hospital affairs and positive nurse–physician relationships were associated with remaining in employment. At a broader level, Chang et al. [12] found that organisational factors accounted for the largest proportion of turnover influences identified across six systematic reviews, including work environment, workload, scheduling, leadership, teamwork and opportunities for continuing education and promotion.

Greater control over working time appears particularly relevant to the attraction of non‐permanent employment. Across the included studies, nurses valued the ability to select shifts, influence when and where they worked and accommodate family responsibilities, further study, other employment or changing personal circumstances [18, 20–23, 41, 51, 61, 64]. This pattern aligns with Emmanuel et al. [67], who found that nurses’ shift preferences were strongly influenced by priorities outside work, including childcare, social relationships, health and recovery. Nurses particularly valued predictable rosters, adequate rest and greater flexibility and control over working time. Muir et al. [13] further found that family obligations were a prominent reason for leaving healthcare employment, particularly among nurses aged 31 to 50 years, and identified rigid scheduling as a potential barrier to maintaining employment alongside caregiving responsibilities.

Employment decisions regarding nonpermanent arrangements, therefore, appear to reflect the fit between nurses’ personal circumstances, preferred working arrangements and the organisational conditions available to them. A nurse could simultaneously be seeking greater scheduling autonomy and moving away from inflexible rostering, inadequate staffing or unsupportive workplace conditions. Financial incentives and clinical variety provided additional attractions in some studies, but neither was uniformly prioritised across the included evidence. This interpretation is consistent with the broader turnover literature, which identifies organisational, individual and work‐life factors as overlapping influences on nurses’ workforce participation rather than isolated determinants [12, 13].

### 4.2. Belonging, Professional Development and Peripheral Workforce Positioning

Greater flexibility in nonpermanent work did not consistently translate into professional inclusion within the clinical teams in which nonpermanent nurses practised. Although control over working arrangements was a prominent attraction of nonpermanent roles, the included studies also described marginalisation, weaker relationships with permanent colleagues, limited team acceptance and differential treatment [19, 20, 22, 52, 53, 58, 59]. Employment arrangements that increase choice over when and where nurses work may, therefore, still position them at the periphery of the relational and informational networks embedded within established clinical teams. More broadly, Telschow and Berg [28] similarly identified ward culture, relationships with permanent staff, orientation and access to support as important to agency nurse integration. Repeated movement between settings may also require nurses to undergo recurrent processes of organisational socialisation, acquiring local knowledge, role expectations and workplace relationships each time they enter a new setting [68].

Choosing nonpermanent work can involve structural features such as mobility and reduced organisational continuity, but these should be distinguished from adverse experiences arising within the workplaces to which nurses are deployed. Travel nursing, for example, involves repeated movement between assignments, unfamiliar clinical environments and the need to adapt to new organisations and teams [57]. Similar features of mobility and organisational transition have also been described in recent travel‐nursing research [69]. However, social exclusion, stereotyping, questioning of professional competence and limited access to development were not uniformly reported across nonpermanent nursing settings [20, 52, 61]. Some nurses described outsider positioning, close scrutiny and reduced opportunities for skill development [52], whereas others reported welcoming colleagues, team acceptance and supportive professional relationships [20, 61]. This variation suggests that such experiences are shaped at least partly by local workplace relationships and organisational practices rather than by nonpermanent employment status alone.

Formal professional development was less visible within the charted evidence compared to the experiential learning illustrated across nonpermanent roles. Nonpermanent nurses gained knowledge and skills through varied clinical exposure, patient care and informal exchange with colleagues, but access to formal education, feedback and structured development was less consistent [21–23, 52, 54, 59, 61]. Experiential learning can strengthen clinical capability when accompanied by structured reflection, feedback and opportunities to apply learning [70]. However, the included studies indicate that access to formal education, feedback and structured professional development was inconsistent for nonpermanent nurses [21–23, 52, 54, 59, 61]. This distinction is also reflected in recent travel‐nursing research, in which mobility contributed to adaptability, confidence and broader career possibilities while potentially weakening continuity and institutional ties [69]. Broader nursing workforce evidence similarly identifies continuing education, career advancement, leadership and workplace support as important organisational influences on retention [12, 71].

Supportive collegial relationships were an important source of both social inclusion and professional support for nonpermanent nurses working across unfamiliar clinical settings [20, 57, 59, 61]. Welcoming colleagues, team acceptance and acknowledgement of contribution were reported alongside marginalisation, outsider positioning and weaker team relationships [20, 22, 61, 62]. Collegial relationships also provided access to local knowledge, feedback and informal learning opportunities, linking workplace inclusion with nurses’ day‐to‐day professional functioning and development [20, 54, 61]. The broader importance of collegial connection is reflected in Pressley et al. [72], who identified mentorship, peer support and supportive leadership as facilitators of professional integration among internationally recruited nurses, although this population differs from the non‐permanent nurses examined in the present review.

Access to professional relationships, feedback and development may also influence the psychological resources nurses draw on when adapting to unfamiliar work environments. Collegial and organisational support was reported to assist non‐permanent nurses navigate unfamiliar clinical settings, whereas inconsistent support and development were associated with uncertainty, isolation and concerns about professional competence [20, 22, 54, 57, 59, 61]. These findings can be interpreted alongside broader nursing research in which autonomy and competence have been conceptualised as psychological resources, while supportive interpersonal relationships have been linked with psychological need satisfaction and professional adaptation [73, 74]. Broader workplace evidence similarly found that self‐efficacy and resilience were associated with lower burnout, with self‐efficacy also associated with greater flourishing [75]. Flexibility may, therefore, increase control over the structure of employment, while professional confidence, perceived competence and connection may be strengthened or undermined by the quality of nurses’ interactions with the teams and organisations in which they work.

Flexibility in working arrangements does not necessarily translate into a fully supported professional experience when access to professional development, recognition and collegial connection is inconsistent. This interpretation is reinforced by Kößler et al. [76], who found no evidence that temporary nurses experienced greater satisfaction of autonomy than matched permanent nurses but identified significantly lower satisfaction of relatedness among temporary nurses. The distinction is important: flexibility enabled nurses to exercise greater choice over when and where they worked [18, 21–23, 61, 64], while their experiences within the workplace were shaped by access to social connection, professional recognition and development opportunities [19, 20, 22, 52, 54, 61]. For nurse leaders, this distinction shifts attention from the inherent mobility of nonpermanent work towards the organisational conditions that determine whether nurses remain professionally connected, recognised and supported.

### 4.3. Implications for Nursing Management

Organisational support for nonpermanent nurses was inconsistent across orientation, information access, collegial support, recognition and professional development, placing several modifiable conditions within the remit of nurse leadership [20–22, 40, 57, 59, 61]. These findings identify organisational priorities for local planning and evaluation; they do not establish the effectiveness of specific interventions. Nonpermanent staffing should, therefore, be incorporated deliberately into workforce planning rather than managed solely as an immediate response to staffing deficits. This does not imply that temporary staffing can substitute for an adequately staffed permanent workforce. In a longitudinal study that analysed a cohort of 626,313 hospital admissions, it was found that temporary nurses partially mitigated the increased mortality associated with low staffing, but risk remained elevated compared with adequate baseline permanent staffing [77].

Nonpermanent nurses’ difficulties in unfamiliar clinical settings frequently reflected limited access to local knowledge, routines and support rather than deficits in underlying professional competence [20, 40, 57, 59, 61]. Griffiths et al. [77] identified unfamiliarity with the care setting as a hypothesised mechanism through which temporary staffing may affect efficiency and continuity, rather than attributing these associations to deficits in nurses’ clinical competence. Wolbers et al. [65] similarly identified access to situated and often tacit knowledge about patients, organisational processes and local practice as important to the effective deployment of experienced but unfamiliar temporary nurses. Orientation should, therefore, prioritise rapid access to unit‐specific routines, equipment, communication and escalation pathways, documentation processes and sources of clinical support. Krzyzewski et al. [78] further support unit‐specific orientation, mentorship, access to essential resources and ongoing feedback when integrating travelling clinical staff.

Integration into established clinical teams was shaped by the quality of collegial relationships and the support available from permanent staff, with experiences ranging from guidance and inclusion to marginalisation and differential treatment [20, 22, 52, 58, 61, 62]. The responsibility for integration should not be transferred informally to permanent nurses who may already be working under constrained staffing conditions. Nurse leaders need to account for the time and relational work required to orient unfamiliar colleagues, maintain clear role expectations and support communication across the team. This includes establishing clear expectations for respectful team behaviour and responding promptly to bullying, stereotyping, exclusion and inequitable treatment, which were reported in some nonpermanent nursing settings [52, 58]. Integration should also be reciprocal. Nonpermanent nurses bring experience from different organisations and clinical settings, and supportive work environments may allow this expertise to contribute to shared learning rather than positioning temporary nurses solely as recipients of support [65].

Formal professional development was less consistently accessible than experiential learning, highlighting the need for explicit organisational responsibility for feedback, continuing education and professional progression [21–23, 54, 59, 61]. Nurse leaders should, therefore, clarify responsibility for feedback, competency development and access to continuing education, particularly where responsibility is divided between a staffing agency and host organisation. Development systems should also recognise existing expertise and provide opportunities for nonpermanent nurses to contribute knowledge gained across clinical settings [53, 54]. Agency nurses in these studies were described as bringing ideas and practices from other units and organisations, creating opportunities for knowledge sharing and competence development among permanent staff. Mobility should, therefore, not be assumed to be incompatible with professional growth, as travel nursing has also been associated with increased adaptability, confidence, expertise and broader career trajectories [69].

Nonpermanent nurses’ professional confidence, perceived competence and connection were linked in their accounts to workplace conditions, including recognition, feedback, collegial support and access to development [20, 22, 52, 59, 61, 62]. Where these conditions were limited, nurses described anxiety or uncertainty, isolation and concerns about maintaining or demonstrating professional competence [52, 59–61]. These patterns suggest that such experiences are shaped partly by organisational conditions, limiting the usefulness of approaches that place primary responsibility on individual resilience. Broader nursing evidence similarly shows that teamwork, recognition, knowledge sharing and opportunities to exercise expertise can support nurses’ experiences of competence and professional meaning, while distrust, limited professional voice and unsafe learning environments can undermine them [79]. Priorities for nurse leaders include approachable clinical support, constructive feedback, recognition of existing expertise, psychologically safe opportunities to ask questions and meaningful participation in team practice.

Reliance on nonpermanent staffing may reflect not only fluctuating workforce demand but also modifiable weaknesses in the conditions of permanent employment, including inflexible rostering, burnout and unsupportive work environments [21, 57, 58, 60, 64]. Ghimire [80] similarly conceptualised agency reliance as both a staffing strategy and an organisational diagnostic, with agency nurses describing movement away from permanent roles characterised by inflexible rostering, bureaucratic burden and unsupportive leadership. Organisational priorities, therefore, include integrating, recognising and supporting nonpermanent nurses while examining whether modifiable features of permanent employment are contributing to continued reliance on contingent staffing.

## 5. Limitations

The main limitations of this review arise from the scope of the search and the synthesis process. Only English language empirical studies were eligible, while grey literature, dissertations, conference abstracts and other nonempirical sources were excluded. Relevant evidence published in other languages, indexed outside the five databases searched or available outside the published empirical literature may, therefore, have been missed. The terminology used to describe nonpermanent nursing also varies considerably across jurisdictions and employment arrangements; despite the use of a broad search strategy, studies using terminology not captured by the search terms may not have been identified.

The qualitative synthesis also involved interpretive decisions that may have influenced category development. Although study selection was undertaken independently by two reviewers, completed data charting was checked by a second reviewer, and the initial extraction and coding approach was developed collaboratively, one reviewer subsequently undertook extraction and coding for the remaining studies. Developing categories were reviewed and discussed by the wider review team, which provided opportunities to examine coding decisions and alternative interpretations, but this process cannot entirely remove the influence of researcher interpretation.

## 6. Future Research Considerations

Future research should address the demographic, geographic, setting, and temporal gaps in the existing evidence on nonpermanent nursing. Studies are needed across less represented healthcare systems and practice settings, including primary care, community care and aged care, and across the range of nonpermanent employment arrangements identified in this review. More detailed examination of age, gender, cultural background, visa or immigration status, caregiving responsibilities and career stage may also clarify whether motivations, professional experiences and support needs differ between groups. Longitudinal research is particularly needed to examine how engagement in nonpermanent work changes over time and its longer term relationships with wellbeing, professional development, career progression, retention and movement between permanent and nonpermanent employment.

The organisational and relational conditions shaping nonpermanent nurses’ experiences also require more direct investigation. Future studies could incorporate the perspectives of nurse managers, educators, permanent colleagues and patients to examine how nonpermanent staffing influences communication, team integration, role expectations and clinical practice. Empirical evaluation of support strategies is also needed, particularly structured and context‐specific orientation, timely access to local information, designated clinical support, feedback mechanisms and equitable access to professional development. Comparative and intervention studies would help establish which approaches improve nurses’ confidence, integration, job satisfaction and sustained engagement rather than relying predominantly on descriptive accounts of unmet support needs.

Greater theoretical and outcome‐focused research could clarify how workplace conditions influence the consequences of nonpermanent employment. The job demands–resources model [81, 82] may provide a useful framework for examining how demands associated with unfamiliar environments, workload and employment insecurity interact with resources such as autonomy, collegial support, information access and development opportunities. Self‐determination theory [83–85] may similarly support investigation of how workplace conditions relate to experiences of autonomy, competence and relatedness. Theory‐informed studies could, therefore, move beyond describing associations towards testing mechanisms and developing targeted interventions [86, 87]. Research should also examine the direct and indirect effects of nonpermanent staffing arrangements on patient safety and quality of care, particularly alongside staffing levels, workforce composition and continuity of care.

## 7. Conclusion

Nonpermanent nursing provides valued flexibility and employment choice, but nurses’ experiences of these roles depend substantially on the conditions in which the work occurs. Across 31 empirical studies, nurses described flexibility, control over working arrangements, financial considerations, varied clinical experience and compatibility with personal circumstances as reasons for choosing or continuing nonpermanent work. Movement into these roles was also influenced by dissatisfaction with aspects of permanent employment, including inflexible rostering, workplace stress, inadequate staffing and limited leadership support. Nonpermanent work could broaden clinical experience and support skill development, yet access to formal education, feedback and career progression was variable and frequently limited.

The professional experience of nonpermanent nurses was strongly shaped by their access to information, collegial relationships, recognition and support within the workplaces in which they were deployed. Working across unfamiliar settings created particular demands when nurses lacked knowledge of local routines, systems and expectations. Experiences of team integration also varied, ranging from supportive collegial relationships to marginalisation, differential treatment and limited recognition of professional expertise. Together, the findings show that employment flexibility does not necessarily translate into a consistently positive professional experience and that the benefits and challenges of nonpermanent work are closely connected to the organisational environment.

Many of the support needs identified across the evidence base are, therefore, amenable to organisational and nursing leadership practices. Structured and context‐specific orientation, timely access to local information and clinical support, clear role expectations, inclusive communication, recognition of professional contribution, and equitable access to feedback and development opportunities emerged as important conditions for supporting nonpermanent nurses. As health services continue to use nonpermanent staffing to supplement workforce capacity, incorporating these nurses deliberately into workforce planning and clinical teams may help preserve the flexibility these arrangements provide while reducing avoidable barriers to confidence, professional development, belonging and sustained engagement.

## Funding

This research received no specific grant from any funding agency in the public, commercial or not‐for‐profit sectors. Open access publishing facilitated by Australian Catholic University, as part of the Wiley ‐ Australian Catholic University agreement via the Council of Australasian University Librarians Australian Catholic University.

## Conflicts of Interest

The authors declare no conflicts of interest.

## Supporting Information

Additional supporting information can be found online in the Supporting Information section.

## Supporting information


**Supporting Information 1** Supplementary File 1 provides the database‐specific search strategies used in this scoping review.

**Supporting Information 2** PRISMA‐ScR checklist.

## Data Availability

This scoping review used data extracted from published studies cited in the article. Study characteristics and summaries of findings are presented in Table 1, and the search strategies are provided in the Supporting Information. No new participant‐level data were collected.
