# Supplementary material for: Nurses’ Experiences, Employment Decisions and Support Needs in Nonpermanent Roles: A Scoping Review
Source: J Nurs Manag. 2026 Oct 28;2026:4509202. doi: 10.1155/jonm/4509202 (PMC13714634; doi:10.1155/jonm/4509202)
Supplement: Supplementary file 1 [file JONM-2026-4509202-s001.docx]

**Supplementary File 1: Search strategies**

| **Database** | **PICo** | **Search terms** |
| --- | --- | --- |
| **CINAHL complete** | **Population** | ( "nurs*" OR "registered nurs*" OR "enrolled nurs*" OR "nurs* workforce" OR “licensed Nurs*” ) AND ( “Casual work” OR "casual employment" OR “agency work” OR "non-permanent work" OR “non-traditional work” OR “non-traditional employment” OR “temporary employment” OR "temporary nurs*" OR "per diem nurs*" OR "agency nurs*" OR "bank nurs*" OR "locum nurs*" OR “travel nurs*” OR "contingent employment" OR “temporary employment” OR “flexible schedul*” ) |
|  | **AND** | |
|  | **Interest** | ( "experience*" OR “work experience*” OR "perception*" OR "decision*" OR "career choice*" OR "employment decision*" OR "job preferenc*" OR "motivat*" OR "barrier*" OR “facilit*” OR "lived experience*" OR “Nurse profession*” OR “Health Profession*” OR “decision making” OR “Career Progress*” OR “career advance*” ) |
|  | **AND** | |
|  | **Context** | ( "hospital*" OR "acute care" OR "healthcare facilit*" OR "nursing home*" OR "aged care" OR "primary care" OR "community health" OR “mental Health” OR “critical care” OR “Health clinic” OR “Clinic” OR “workplace” ) |
| **Health source: Nursing/Academic** | **Population** | ( "nurs*" OR "registered nurs*" OR "enrolled nurs*" OR "nurs* workforce" OR “licensed Nurs*” ) AND ( “Casual work” OR "casual employment" OR “agency work” OR "non-permanent work" OR “non-traditional work” OR “non-traditional employment” OR “temporary employment” OR "temporary nurs*" OR "per diem nurs*" OR "agency nurs*" OR "bank nurs*" OR "locum nurs*" OR “travel nurs*” OR "contingent employment" OR “temporary employment” OR “flexible schedul*” ) |
|  | **AND** | |
|  | **Interest** | ( "experience*" OR “work experience*” OR "perception*" OR "decision*" OR "career choice*" OR "employment decision*" OR "job preferenc*" OR "motivat*" OR "barrier*" OR “facilit*” OR "lived experience*" OR “Nurse profession*” OR “Health Profession*” OR “decision making” OR “Career Progress*” OR “career advance*” ) |
|  | **AND** | |
|  | **Context** | ( "hospital*" OR "acute care" OR "healthcare facilit*" OR "nursing home*" OR "aged care" OR "primary care" OR "community health" OR “mental Health” OR “critical care” OR “Health clinic” OR “Clinic” OR “workplace” ) |
| **MEDLINE complete** | **Population** | ( "nurs*" OR "registered nurs*" OR "enrolled nurs*" OR "nurs* workforce" OR “licensed Nurs*” ) AND ( “Casual work” OR "casual employment" OR “agency work” OR "non-permanent work" OR “non-traditional work” OR “non-traditional employment” OR “temporary employment” OR "temporary nurs*" OR "per diem nurs*" OR "agency nurs*" OR "bank nurs*" OR "locum nurs*" OR “travel nurs*” OR "contingent employment" OR “temporary employment” OR “flexible schedul*” ) |
|  | **AND** | |
|  | **Interest** | ( "experience*" OR “work experience*” OR "perception*" OR "decision*" OR "career choice*" OR "employment decision*" OR "job preferenc*" OR "motivat*" OR "barrier*" OR “facilit*” OR "lived experience*" OR “Nurse profession*” OR “Health Profession*” OR “decision making” OR “Career Progress*” OR “career advance*” ) |
|  | **AND** | |
|  | **Context** | ( "hospital*" OR "acute care" OR "healthcare facilit*" OR "nursing home*" OR "aged care" OR "primary care" OR "community health" OR “mental Health” OR “critical care” OR “Health clinic” OR “Clinic” OR “workplace” ) |
| **Psychinfo®** | **Population** | ( "nurs*" OR "registered nurs*" OR "enrolled nurs*" OR "nurs* workforce" OR “licensed Nurs*” ) AND ( “Casual work” OR "casual employment" OR “agency work” OR "non-permanent work" OR “non-traditional work” OR “non-traditional employment” OR “temporary employment” OR "temporary nurs*" OR "per diem nurs*" OR "agency nurs*" OR "bank nurs*" OR "locum nurs*" OR “travel nurs*” OR "contingent employment" OR “temporary employment” OR “flexible schedul*” ) |
|  | **AND** | |
|  | **Interest** | ( "experience*" OR “work experience*” OR "perception*" OR "decision*" OR "career choice*" OR "employment decision*" OR "job preferenc*" OR "motivat*" OR "barrier*" OR “facilit*” OR "lived experience*" OR “Nurse profession*” OR “Health Profession*” OR “decision making” OR “Career Progress*” OR “career advance*” ) |
|  | **AND** | |
|  | **Context** | ( "hospital*" OR "acute care" OR "healthcare facilit*" OR "nursing home*" OR "aged care" OR "primary care" OR "community health" OR “mental Health” OR “critical care” OR “Health clinic” OR “Clinic” OR “workplace” ) |
| **Scopus** | **Population** | ( TITLE-ABS-KEY ( "nurs*" OR "registered nurs*" OR "enrolled nurs*" OR "nurs* workforce" OR "licensed Nurs*" ) ) |
|  |  | AND |
|  |  | ( ( TITLE-ABS-KEY ( "non-permanent employment" ) ) OR ( TITLE-ABS-KEY ( "flexible schedul*" ) ) OR ( TITLE-ABS-KEY ( "temporary employment" ) ) OR ( TITLE-ABS-KEY ( "contingent employment" ) ) OR ( TITLE-ABS-KEY ( "travel nurs*" ) ) OR ( TITLE-ABS-KEY ( "locum nurs*" ) ) OR ( TITLE-ABS-KEY ( "bank nurs*" ) ) OR ( TITLE-ABS-KEY ( "agency nurs*" ) ) OR ( TITLE-ABS-KEY ( "per diem nurs*" ) ) OR ( TITLE-ABS-KEY ( "temporary nurs*" ) ) OR ( TITLE-ABS-KEY ( "non-traditional work" ) ) OR ( TITLE-ABS-KEY ( "non-traditional employment" ) ) OR ( TITLE-ABS-KEY ( "non-permanent work" ) ) OR ( TITLE-ABS-KEY ( "casual employment" ) ) OR ( TITLE-ABS-KEY ( "agency work" ) ) OR ( TITLE-ABS-KEY ( "Casual work" ) ) ) ) |
|  | **AND** | |
|  | **Interest** | ( ( TITLE-ABS-KEY ( "career advanc*" ) ) OR ( TITLE-ABS-KEY ( "Career Progress*" ) ) OR ( TITLE-ABS-KEY ( "decision making" ) ) OR ( TITLE-ABS-KEY ( "Healthcare Profession*" ) ) OR ( TITLE-ABS-KEY ( "Nurse profession*" ) ) OR ( TITLE-ABS-KEY ( "lived experience*" ) ) OR ( TITLE-ABS-KEY ( "facilit*" ) ) OR ( TITLE-ABS-KEY ( "barrier*" ) ) OR ( TITLE-ABS-KEY ( "motivat*" ) ) OR ( TITLE-ABS-KEY ( "job preference*" ) ) OR ( TITLE-ABS-KEY ( "employment decision*" ) ) OR ( TITLE-ABS-KEY ( "career choice*" ) ) OR ( TITLE-ABS-KEY ( "decision*" ) ) OR ( TITLE-ABS-KEY ( "perception*" ) ) OR ( TITLE-ABS-KEY ( "experience*" ) ) ) |
|  | **AND** | |
|  | **Context** | ( "hospital*" OR "acute care" OR "healthcare facilit*" OR "nursing home*" OR "aged care" OR "primary care" OR "community health" OR “mental Health” OR “critical care” OR “Health clinic” OR “Clinic” OR “workplace” ) |
